# Aging‐associated dysregulation of homeostatic immune response termination (and not initiation)

**DOI:** 10.1111/acel.12589

**Published:** 2017-03-30

**Authors:** Goutham Pattabiraman, Karol Palasiewicz, John P. Galvin, David S. Ucker

**Affiliations:** ^1^ Department of Microbiology and ImmunologyUniversity of Illinois College of MedicineChicagoIL60612USA; ^2^ Present address: Department of ImmunologyUniversity of Connecticut Health CenterFarmingtonCT06030USA; ^3^ Present address: Department of Medicine (Division of Hematology/Oncology)Robert H. Lurie Cancer CenterNorthwestern UniversityChicagoIL60611USA

**Keywords:** aging, apoptosis, homeostasis, immunosenescence, innate immunity, macrophages

## Abstract

Immunosenescence is a state of unbalanced immune responsiveness, characterized by a diverse repertoire of seemingly discreet and paradoxical alterations in all aspects of immunity arising in an aging‐associated manner. We asked whether aging‐associated alterations in the ability of apoptotic cells to elicit immunomodulatory responses (innate apoptotic immunity; IAI) or in IAI responses themselves might underlie the confounding aging‐associated anomalies of immunosenescence. We explored this question by examining, as a function of animal age, responsiveness of murine macrophages on the single cell level. We monitored the expression of pro‐ and anti‐inflammatory cytokines cytofluorimetrically in response to pro‐inflammatory Toll‐like receptor (TLR) stimulation and anti‐inflammatory treatment with apoptotic cells. While we found no alterations with age in the potency of apoptotic cells or in the initiation and magnitude of IAI responses, we did identify a cell‐intrinsic deficiency in anti‐inflammatory IAI response termination linked with age and preceding manifestations of immunosenescence. Further, we found that an aging‐associated deficiency in response termination also is evident following TLR stimulation. These surprising observations reveal that a loss of homeostatic immune control with animal age results from the dysregulation of response termination (as distinct from response initiation) and is exerted on the level of transcription. We suggest that, with advancing age, cells become locked into relatively longer‐lived response states. Aging‐associated immune dysfunctions may reflect a diminution in the cellular nimbleness of immune responsiveness.

**Abbreviations**
IAI: Innate Apoptotic Immunity
IL: Interleukin
LPS: Lipopolysaccharide
MFI: Mean Fluorescence Intensity
PE: Phycoerythrin
RT–qPCR: Reverse Transcription–quantitative Polymerase Chain Reaction
TLR: Toll‐like Receptor
TNFα: Tumor Necrosis Factor‐α

## Introduction

Aging is associated with waning of immune function (Price & Makinodan, 1972; Thoman & Weigle, 1989), and this ‘immunosenescence’ has been hypothesized to underlie causally many of the pathologies of aging (Walford, 1969). Immunosenescence is not limited to humans; its attributes have been characterized in numerous primate and other mammalian species, and even in birds (Haussmann *et al*., 2005). Declines with increasing age of T‐lymphocyte function and T‐cell repertoire (Zhang *et al*., 2002), diminution of B‐lymphocyte activity, including T‐independent activity (Gerbase‐DeLima *et al*., 1974), and reduced responsiveness to vaccination [e.g., influenza immunization (Toapanta & Ross, 2009)], have been well described (Inamizu *et al*., 1985). Beyond lymphocytes, functional changes in the myeloid cell compartment also have been recognized; data suggest that these declines are equally consequential (Inamizu *et al*., 1985). For example, the decline in the antigen presenting and costimulatory functions of ‘accessory’ cells (including macrophages) may underlie the aging‐associated reduction in antigen‐specific immune responsiveness (Bondada *et al*., 2000).

Increases in circulating and localized levels of inflammatory cytokines – even in the absence of overt infection – also are associated with advanced age (Thoman & Weigle, 1989; Ershler, 1993; Bruunsgaard *et al*., 1999; Walston *et al*., 2002; Ferrucci *et al*., 2005; Jeon *et al*., 2012). The simple view of the aged immune system as one of heightened inflammatory status may not represent accurately the situation, however. Certainly, the aging‐associated elevation of levels of circulating autoantibodies [rheumatoid factors, including antinuclear antibodies (Walford, 1969)], especially contemporaneous with a waning of antigen‐specific responsiveness represents a confounding complexity. An alternative characterization suggests that the state of the aged immune system is not unilaterally pro‐inflammatory, but rather dysregulated both with respect to pro‐ and anti‐inflammatory factors. Notably, the pattern of age‐related increase observed with TNFα and Interleukin [IL]‐6 levels is not matched for other pro‐inflammatory cytokines, including IL‐1β, while levels of anti‐inflammatory cytokines, such as IL‐10, also are altered (Saurwein‐Teissl *et al*., 2000; Ferrucci *et al*., 2005; Jeon *et al*., 2012). Although a causal role for elevated cytokine levels in aging‐associated pathologies has not been established, the analysis of genetic polymorphisms in the IL‐6 promoter has implicated enhanced pro‐inflammatory cytokine expression as predictive, at least, of aging‐associated cardiovascular disease (Jenny *et al*., 2002). Complementarily, an IL‐10 promoter polymorphism associated with increased anti‐inflammatory cytokine expression is predictive of healthy aging (Lio *et al*., 2004). Intriguingly, some studies have suggested that the dynamics of cytokine production (and dampening) may be altered with age (Krabbe *et al*., 2004; Shaw *et al*., 2010). It may be that the immunosenescent state can be described more comprehensively as ‘imbalanced’ (Saurwein‐Teissl *et al*., 2000).

Macrophages are sentinels of stress and immunological challenge. They are heterogeneous and flexibly responsive, and they respond nimbly as central effectors of innate immunity. This critical role has prompted numerous studies regarding the involvement of macrophages in aging‐associated immune anomalies. This work has led to the identification of several intrinsic aging‐associated macrophage alterations. For example, aging‐associated reductions in their production of reactive oxygen and nitrogen species (Chen *et al*., 1991; Plackett *et al*., 2004) and in their antitumor activity (Wallace *et al*., 1995) have been noted. In addition, impaired intracellular signal transduction [especially via the JAK/Stat and mitogen‐activated protein kinase (MAPK) pathways] with age has been reported (Yoon *et al*., 2004; Chelvarajan *et al*., 2006; Panda *et al*., 2009). Studies of inflammatory cytokine production by macrophages, in contrast, have not revealed a definitive aging‐associated change. Our studies of murine macrophages have demonstrated that inflammatory responsiveness elicited via Toll‐like receptor (TLR) engagement is not altered generally with age (Pattabiraman *et al*., 2016). Other work, with human and rodent macrophages, has documented diminished responsiveness to certain innate immune stimuli in some (Renshaw *et al*., 2002; Boehmer *et al*., 2005; Chelvarajan *et al*., 2005; van Duin *et al*., 2007), but not all (Ahluwalia *et al*., 2001) cases, and caveats regarding several of those studies call into question their particular conclusions (Pattabiraman *et al*., 2016). It is clear that results from *ex vivo* and *in vitro* studies do not comport with the view of aging‐associated immune dysregulation characterized simply as heightened inflammatory status. Similarly, the suggestion that isolated macrophage (and monocyte) phenotypes are skewed with age toward an anti‐inflammatory (alternatively activated, ‘M2‐like’) state (Boehmer *et al*., 2005) is not readily consistent with *in vivo* observations.

Our recent studies have highlighted the remarkable effects of apoptotic cells on immunological (including inflammatory) responsiveness of macrophages and other cells with which they interact (Birge & Ucker, 2008). The process of specific apoptotic cell recognition and response represents a ubiquitous and unconventional innate immunity (‘innate apoptotic immunity’, IAI) that discriminates effete from viable cells, without regard to self, and that potently modulates responsiveness (Cvetanovic *et al*., 2006). The modulatory activity of the apoptotic corpse, representing a gain of function acquired during the physiological cell death process, is manifest both as the immediate‐early induction of anti‐inflammatory cytokine gene transcription and inhibition of pro‐inflammatory cytokine gene transcription within the responding cell (Cvetanovic & Ucker, 2004). It is exerted upon binding, independent of TLR signaling, subsequent engulfment, and soluble factor involvement (Cvetanovic & Ucker, 2004; Cvetanovic *et al*., 2006; Ucker, 2009). In this latter context, the apoptotic effect can be viewed as the induction of a state refractory to pro‐inflammatory responsiveness. Several molecules that function intracellularly as enzymes involved in glycolysis become externalized on the surface of apoptotic cells and function as apoptotic recognition determinants (Ucker *et al*., 2012).

A failure of IAI has been linked to inflammatory and autoimmune pathology, including systemic lupus erythematosus (SLE) and rheumatic diseases (Savill *et al*., 2002; Birge & Ucker, 2008). The anti‐inflammatory M2‐like behavior of macrophages elicited by apoptotic cells is a dynamic and transient response, and not a stable phenotypic skewing or polarization. In this sense, IAI can be viewed as a resolving mechanism to restore homeostasis by dampening immune responsiveness. Given the unbalanced nature of the aged immune system, we asked whether the ability of apoptotic cells to elicit IAI responses or IAI responsiveness itself (and particularly the dynamics of that response) might be perturbed with advancing age and underlie immunosenescent dysregulation.

We tested our hypothesis in mice, examining macrophage responses specifically. Extensive analyses documented an absence of aging‐associated alterations in the immunomodulatory potency of apoptotic cells or in the magnitude of IAI responsiveness and confirmed our previous findings of an absence of aging‐associated alterations in the magnitude of TLR responses (Pattabiraman *et al*., 2016). In contrast to end‐point measurements, however, kinetic analyses revealed significant differences in the course of responsiveness. While IAI and TLR‐dependent responses ensue rapidly and are dampened swiftly in macrophages from young adult animals, there appears to be a general deficit in both anti‐ and pro‐inflammatory response termination with advancing age. We interpret these observations to suggest that homeostatic restoration and, consequently, the flexibility with which macrophages can respond to distinct stimuli becomes limited in an aging‐associated manner. We speculate that this alteration in the nimbleness with which macrophages are able to alter their responsiveness may be evinced especially as a reduced ability to dampen pro‐inflammatory responses, and may be of causal significance to the imbalanced immunosenescent state.

## Results

### The magnitude of macrophage IAI responsiveness is unaltered with age

**Figure 1:**
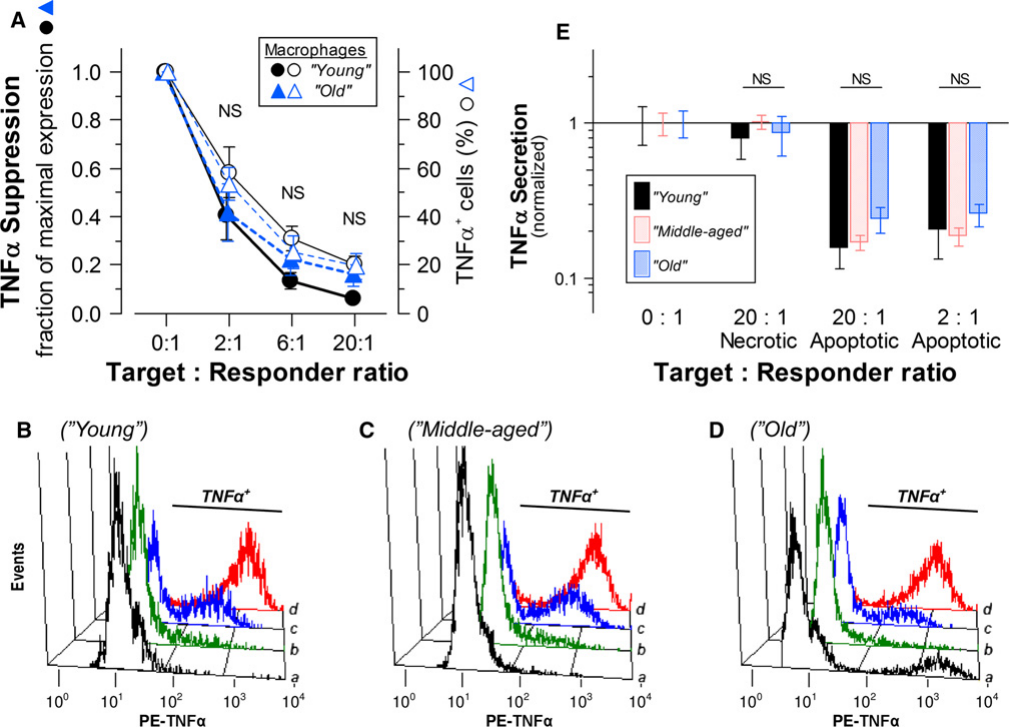
Aging does not alter the magnitude of intrinsic macrophage responsiveness to apoptotic cells – analysis of TNFα expression. (A) The magnitude of apoptotic suppression of TNFα expression in C57BL/6 macrophages isolated from mice of 2–3 months of age (‘young’, 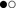) and mice of 24–25 months of age (‘old’, 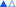) was determined. Elicited peritoneal macrophages from individual mice within each age cohort were cultured without or with apoptotic targets (splenocytes prepared from ‘young’ syngeneic mice, treated with staurosporine) at the indicated target: responder (T: R) ratio for 2 h before the addition of LPS (5 ng mL^−1^) for another 5 h (Target cells were not removed.) Brefeldin A was included during the final 3 h of incubation. Results (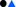) are calculated from the mean fluorescence intensity (MFI) values of gated F4/80^+^ macrophages obtained cytofluorimetrically following intracellular immunostaining with a phycoerythrin (PE)‐conjugated TNFα‐specific antibody (MFI_PE_) and are normalized with respect to the maximum stimulated level of intracellular TNFα (MFI_PE__(_ _LPS__‐Stimulated control)_). In addition, ‘background’ MFI values (MFI_PE__(Unstimulated control)_) are subtracted from experimental values. Calculations were made using the following formula: {MFI_PE__(Sample)_ − MFI_PE__(Unstimulated control)_}/{MFI_PE__(_ _LPS__‐Stimulated control)_ − MFI_PE__(Unstimulated control)_}. Alternatively, results (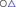) are expressed as the fraction of all F4/80^+^ cells that were TNFα‐positive by intracellular cytokine staining (see next). There were no statistically significant differences in the fractions of unstimulated cells that stained as TNFα positive among cell populations from mice of different ages. The data presented are compiled from the analysis of macrophages from 8 individual mice within each age cohort. Examples of the primary cytofluorimetric data are presented in Panels B–D. There were no statistically significant differences (NS:* Ρ* > 0.05) between age groups by either analytical method, as calculated by Student's *t*‐test. (B–D) Representative examples of the cytofluorimetric analysis of apoptotic suppression of LPS‐induced TNFα expression in viable F4/80^+^ elicited peritoneal macrophages from individual ♀ C57BL/6 mice of different ages are presented. (B) Macrophages from a 3‐month‐old (‘young’) mouse: (A) unstimulated, MFI_PE_ = 22.33; (B) LPS (5 ng mL^−1^) and apoptotic targets (as above; T: R = 20: 1), MFI_PE_ = 26.23; (C) LPS and apoptotic targets (T: R = 2: 1), MFI_PE_ = 66.74; (D) LPS alone, MFI_PE_ = 980.38. (C) Macrophages from a 15‐month‐old (‘middle‐aged’) mouse: (A) unstimulated, MFI_PE_ = 19.78; (B) LPS and apoptotic targets (T: R = 20: 1), MFI_PE_ = 36.26; (C) LPS and apoptotic targets (T: R = 2: 1), MFI_PE_ = 109.75; (D) LPS alone, MFI_PE_ = 742.22. (D) Macrophages from a 24‐month‐old (‘old’) mouse: (A) unstimulated, MFI_PE_ = 28.58; (B) LPS and apoptotic targets (T: R = 20: 1), MFI_PE_ = 17.97; (C) LPS and apoptotic targets (T: R = 2: 1), MFI_PE_ = 29.42; (D) LPS alone, MFI_PE_ = 611.70. (E) TNFα secretion by cultured macrophages from ‘young’ (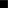), ‘middle‐aged’ (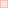), and ‘old’ (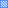) C57BL/6 mice after 20 h of incubation with target cells, as indicated, was determined by multiplex immunoassays. Compiled data are normalized within each age cohort. Data are normalized with respect to cultures stimulated with LPS alone. There were no statistically significant differences (NS:* Ρ* > 0.05) among age groups with respect to modulation of TNFα secretion, as calculated by one‐way ANOVA.

The attenuation of pro‐inflammatory cytokine expression (i.e., the induction of an immunosuppressed state refractory to pro‐inflammatory stimulation) is one of the most dramatic manifestations of IAI. We treated elicited peritoneal macrophages with *E. coli* lipopolysaccharide (LPS) to set the stage for this analysis. We evaluated intrinsic macrophage responses quantitatively on the level of the single cell, using multiparameter cytofluorimetric methods (see Experimental procedures). This is represented in Fig. 1A as a diminution in the level of intracellular TNFα dependent on the ‘dose’ of syngeneic apoptotic cells [the number of apoptotic target cells per responder macrophage (T: R ratio)]. We examined macrophages taken from C57BL/6 mice of discreet ages spanning their normal adult lifespan: young adults (2–3 months of age, ‘young’), middle‐aged adults (15 months of age, ‘middle‐aged’), and older adults nearing the end of mean lifespan (24–25 months of age; ‘old’). We found no evidence of any aging‐associated alteration in the magnitude of responsiveness to apoptotic cells.

The data in Fig. 1A document that the potency of apoptotic cell‐mediated suppression is profound and independent of animal age. Calculating suppression based on TNFα‐specific mean fluorescence intensity (MFI) values from multiple experiments, half‐maximal suppression occurs at a ratio of about two apoptotic splenocyte targets per macrophage (2.3 ± 0.3: 1). Primary cytofluorimetric data are exemplified in Fig. 1B–D. It is clear that there is no statistically significant difference in the magnitude or dose dependency of responsiveness between macrophages from ‘young’ and ‘old’ mice; results with macrophages from ‘middle‐aged’ mice also were indistinguishable (see Fig. 1C and below). Analogous analyses of IAI responses of macrophages from Balb/cBy mice gave entirely comparable results (Fig. S1, Supporting information).

Parallel analyses of IAI responsiveness of macrophages on the population level assessed the secretion of TNFα into culture supernatants (Fig. 1E). These analyses, although less probative of intrinsic cellular issues and involving longer incubations to facilitate the accumulation of readily measurable cytokine levels (which also mitigate target dosage effects), gave entirely consistent results. We observed no evidence of any aging‐associated alteration in the magnitude of IAI responsiveness. These data additionally confirm that, among dead cells, immunosuppressive activity resides uniquely with apoptotic (and not necrotic) targets [Figs 1E and S1, Supporting information; see (Cvetanovic & Ucker, 2004; Cvetanovic *et al*., 2006)].

The mice from which these macrophages were taken did, indeed, exhibit manifestations of immunosenescence, including aging‐associated alterations in cytokine levels. The aging‐associated changes in serum levels of cytokines that we observed (Fig. S2, Supporting information), which are completely in accord with patterns described previously (Jeon *et al*., 2012; Ko *et al*., 2012), cannot be characterized simply as unilaterally pro‐ (or anti‐) inflammatory, reinforcing the notion of an aging‐associated immune ‘imbalance’ (Saurwein‐Teissl *et al*., 2000). We observed other aging‐associated changes, including altered susceptibility to the bacterial pathogen *Listeria monocytogenes* and increases in the frequency of CD44^hi^ T lymphocytes in older mice (Pattabiraman *et al*., 2016).

### Further characterization of the aging‐independent magnitude of macrophage IAI responsiveness

A closer look at the primary cytofluorimetric data (Fig. 1B–D) reveals a conspicuous ‘all‐or‐nothing’ pattern for apoptotic suppression on the level of the single macrophage. TNFα expression and, correspondingly, apoptotic suppression are essentially bimodal within a population: Cells manifest either of two alternative states for TNFα expression. This is most clearly evident in cases where apoptotic suppression is less than complete (i.e., at the lower of the T: R ratios shown in Fig. 1B–D). The resulting macrophages are either positive (within a normally distributed fluorescence intensity range) or negative for TNFα expression; substantial macrophage subpopulations exhibiting intermediate levels of TNFα expression are absent. There were no statistically significant differences in basal or maximal TNFα expression levels between cells from mice of different ages, nor in the fractions of unstimulated cells that stained as TNFα positive between age cohorts. Apoptotic suppression appears as an absolute effect, involving the complete repression of pro‐inflammatory cytokine expression in affected cells. This digital behavior parallels the bimodal response we have observed following TLR stimulation (Pattabiraman *et al*., 2016). Recalculating apoptotic suppression in terms of the fraction of TNFα‐positive cells (Fig. 1A) again indicates that the magnitude of IAI is unaffected by animal age. We also have shown previously that contact with apoptotic cells (or apoptotic membrane determinants) is sufficient to trigger IAI responses; engulfment is not required (Cvetanovic & Ucker, 2004; Cvetanovic *et al*., 2006; Ucker *et al*., 2012). This remains the case independent of animal age (Fig. S3, Supporting information). At low T: R ratios, some responders may not make [sufficient] contact with targets and remain unsuppressed.

We obtained very similar results from our analyses of unelicited peritoneal and splenic macrophages, and from experiments using other pro‐inflammatory stimuli (data not shown). In addition, we found that other apoptotic targets (derived from primary cells of other tissues, established cell lines, and/or induced to die with different suicidal stimuli) were comparably effective, although specific suppressive potency varies among apoptotic cell types (see, e.g., Figs S1 and S4, Supporting information; note that a higher number of apoptotic thymocytes (5.4 ± 0.2) are needed to achieve half‐maximal suppression relative to larger apoptotic splenocytes). In no case did we observe general aging‐associated differences in the magnitude of macrophage responsiveness to pro‐inflammatory stimulation or apoptotic suppression.

**Figure 2:**
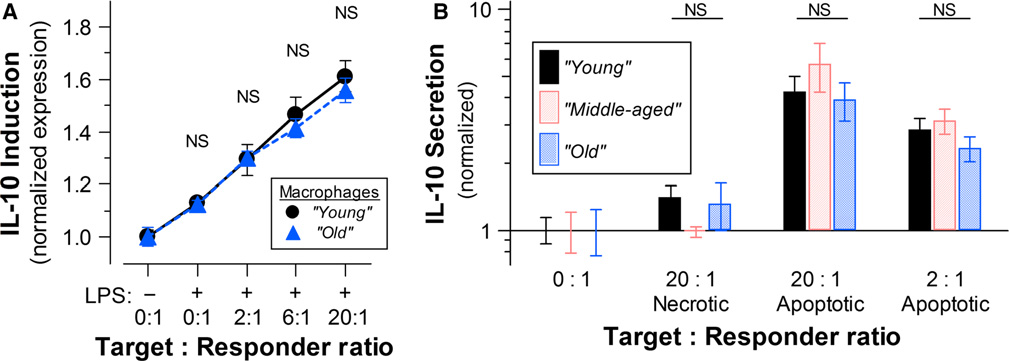
Aging does not alter the magnitude of intrinsic macrophage responsiveness to apoptotic cells – analysis of IL‐10 expression. (A) The magnitude of apoptotic induction of IL‐10 expression in C57BL/6 macrophages isolated from ‘young’ (•) and ‘old’ (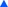) mice was determined. Elicited peritoneal macrophages from individual mice within each age cohort were cultured as indicated with apoptotic targets (as in Fig. 1A) at the indicated T: R ratio and/or LPS; Brefeldin A was included during the last 3 h (Target cells were not removed.) Results are calculated from the MFI values obtained cytofluorimetrically following intracellular immunostaining with an IL‐10‐specific antibody coupled to a PE/cyanine 7 [PE/Cy7] conjugated fluor and are normalized with respect to the levels of intracellular IL‐10 in unstimulated cells. The data presented are compiled from the results of the analysis of macrophages from 6 individual mice within each age cohort. There were no statistically significant differences (NS:* Ρ* > 0.05) between age groups, as calculated by Student's *t*‐test. (B) IL‐10 secretion by cultured macrophages from ‘young’ (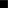), ‘middle‐aged’ (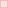) and ‘old’ (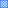) C57BL/6 mice after 20 h of incubation with target cells, as indicated, was determined by multiplex immunoassays. Compiled data are normalized within each age cohort. Data are normalized with respect to unstimulated cultures. The data presented are compiled from the results of the analysis of macrophages from 6 individual mice within each age cohort. There were no statistically significant differences (NS:* Ρ* > 0.05) among age groups with respect to modulation of IL‐10 secretion, as calculated by one‐way ANOVA.

Complementary to their suppression of pro‐inflammatory cytokine gene expression (Cvetanovic & Ucker, 2004; Cvetanovic *et al*., 2006), apoptotic cells trigger the induction of expression of genes encoding anti‐inflammatory cytokines, such as IL‐10 (Fig. 2A). Apoptotic cells stimulate the expression of IL‐10 independently of LPS stimulation, although IL‐10 expression is enhanced by LPS (Fig. 2A). The magnitude of the IL‐10 response assessed in this way is considerably smaller than that for TNFα (Fig. 1A). While IL‐10 induction on the level of transcription in response to apoptotic targets is as rapid as the repression of TNFα transcription, IL‐10 translation and secretion are substantially slower and less efficient (data not shown). Consequently, at the early time point assayed in these experiments (5 h), which captures the dynamics of TNFα modulation, IL‐10 expression has yet to peak, and the induction effected is modest.

**Figure 3:**
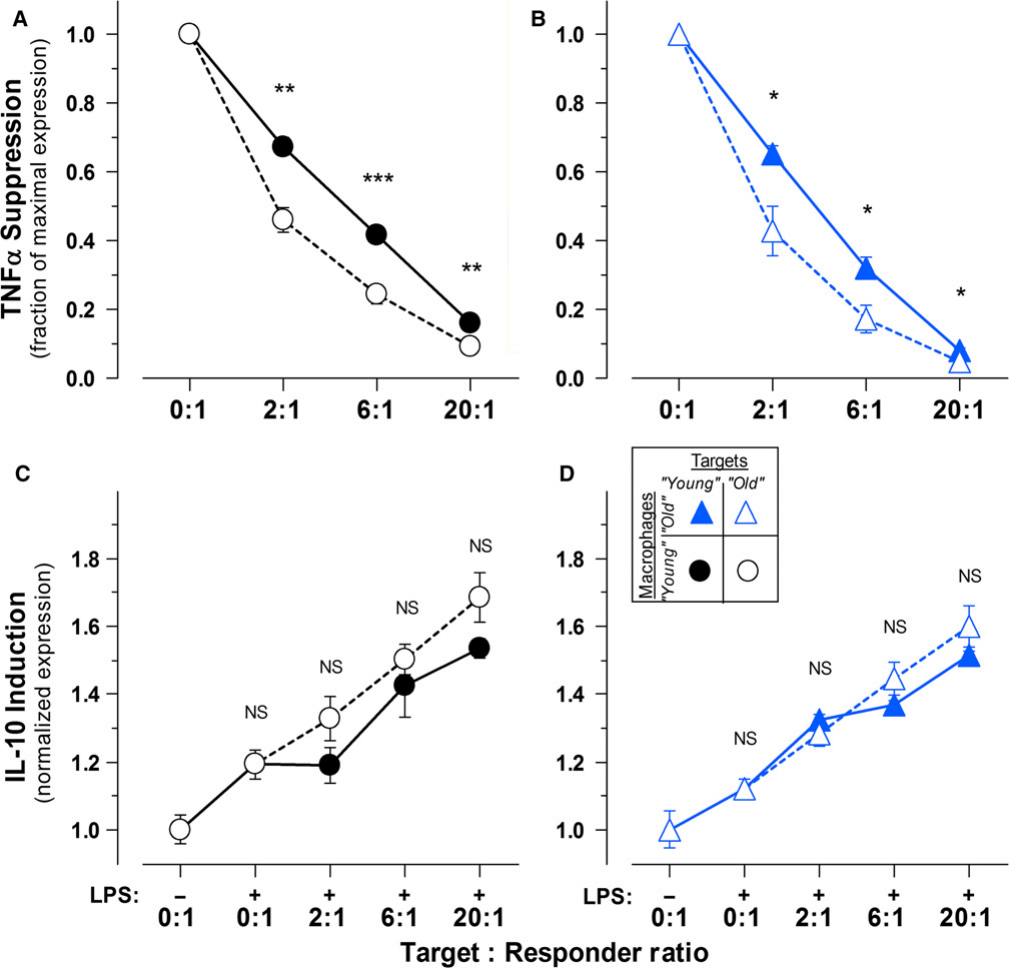
The potency of apoptotic cells to elicit IAI responses is not altered with age. The immunomodulatory activities of apoptotic targets derived from C57BL/6 mice of distinct ages were determined. Apoptotic suppression of TNFα expression was evaluated in syngeneic macrophages isolated from ‘young’ (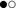; A) and ‘old’ (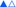; B) mice, as in Fig. 1A. Apoptotic induction of IL‐10 expression in syngeneic macrophages isolated from ‘young’ (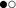; C) and ‘old’ (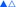; D) mice was determined as in Fig. 2A. Elicited peritoneal macrophages from individual mice of each age cohort were cultured with apoptotic targets (splenocytes prepared from ‘young’ [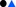] or ‘old’ [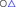] syngeneic mice, and treated with staurosporine) at the indicated T: R ratio. (Target cells were not removed.) The data presented are compiled from the results of the analysis of macrophages from 4 individual mice within each age cohort. The significance of differences between age groups, as calculated by Student's *t*‐test, is indicated (NS:* Ρ* > 0.05; *Ρ ≤ 0.05; **Ρ ≤ 0.01; ***Ρ ≤ 0.001).

With respect to IL‐10 induction, we again observed no differences as a function of animal age in the magnitude of macrophage responsiveness to apoptotic targets. The data in Fig. 2A demonstrate that macrophages from ‘young’ and ‘old’ mice respond identically; the responses of macrophages from ‘middle‐aged’ mice also were indistinguishable (see Fig. 2B). The secretion of IL‐10 into culture supernatants (Fig. 2B) provides another measure of IAI responsiveness of macrophages on the population level. Again, as shown in Fig. 2B, no aging‐associated alteration in the magnitude of macrophage responsiveness is evident. Additionally, as seen both with the suppression of TNFα expression (Fig. 3A,B) and the induction of IL‐10 expression (Fig. 3C,D), apoptotic cells from older animals are no less potent than those from ‘young’ animals in eliciting IAI responses. The biological significance of the modest, albeit statistically significant, aging‐associated augmentation in apoptotic target dose‐dependent response modulation of TNFα (but not IL‐10) expression is not clear.

It is striking that the intrinsic recognition and phagocytic activity of peritoneal macrophages for dead cells is not altered with animal age (Fig. S5, Supporting information). This contrasts with varied reports of aging‐associated alterations in the phagocytic activity of macrophages (Makinodan *et al*., 1971; Chen *et al*., 1991; Linehan *et al*., 2014). While macrophages discriminate apoptotic from necrotic targets with respect to the triggering of IAI responses (Figs 1E and 2B, Figs S1 and S4, Supporting information), both types of dead cells are internalized comparably [Fig. S5, Supporting information; see (Cocco & Ucker, 2001)].

### Impaired macrophage IAI response termination with age

We evaluated the impact of aging on the dynamics of immunosuppression induced in macrophages by apoptotic targets. In distinction from the experiments depicted in Fig. 1A, in which we treated macrophages with a pro‐inflammatory stimulus in the uninterrupted presence of apoptotic cells, we explored the ability of macrophages isolated from mice of different ages to recover responsiveness to TLR‐dependent challenge at varying times following lengthy apoptotic cell interactions.

**Figure 4:**
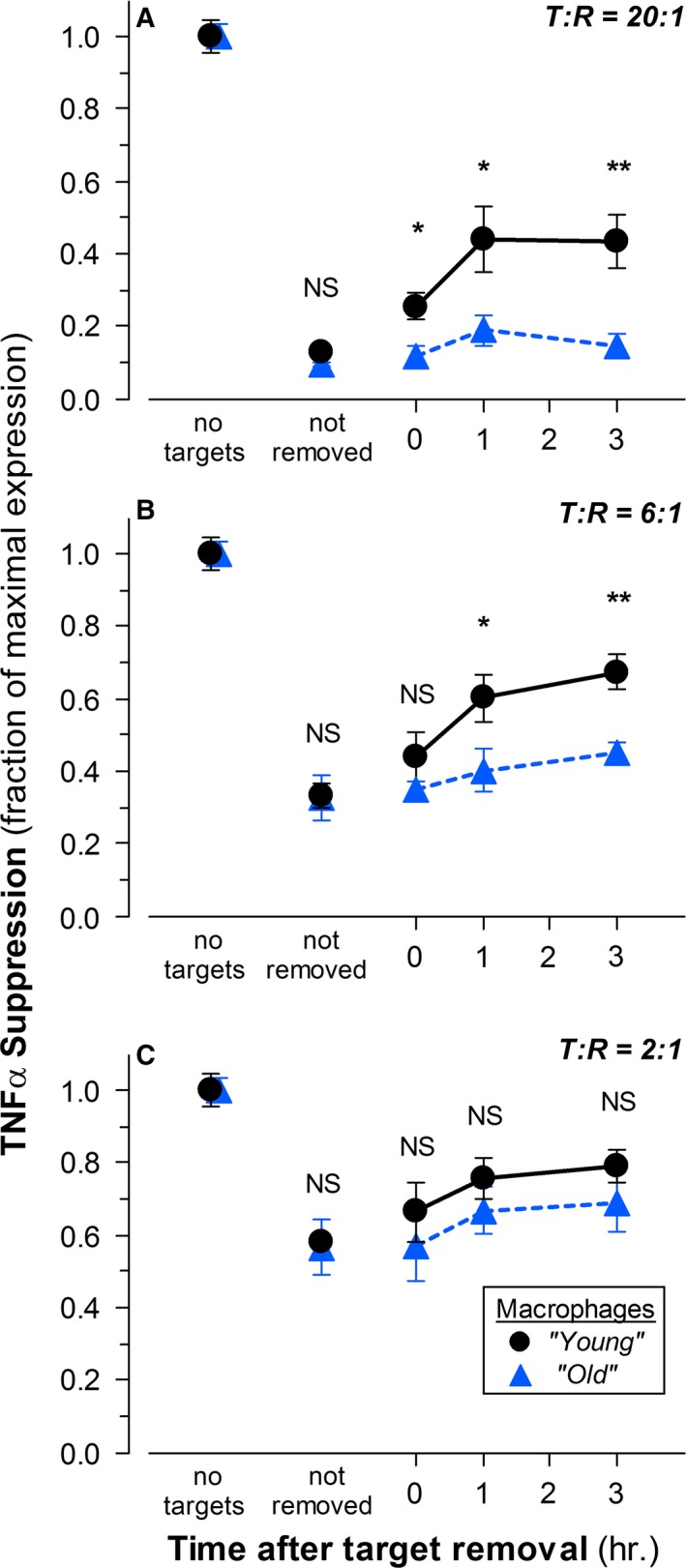
Macrophage IAI response termination is impaired with age. The extent of apoptotic suppression of TNFα expression in elicited peritoneal macrophages isolated from ‘young’ (•) and ‘old’ (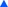) C57BL/6 mice was determined as in Fig. 1A. Macrophages were stimulated with LPS in the absence of apoptotic targets (‘no targets’) and following 20 h of interaction with apoptotic targets at T: R ratios of 20: 1 (A), 6: 1 (B), and 2: 1 (C). Targets remained throughout the subsequent incubation (‘not removed’) or were removed from macrophages by extensive washing before addition of LPS at the indicated times. In all cases, incubation with LPS (5 ng mL^−1^) was for 5 h. Brefeldin A was included during the last 3 h. The data presented are compiled from the results of the analysis of macrophages from eight individual mice within each age cohort. The significance of differences between age groups, as calculated by Student's *t*‐test, is indicated (NS:* Ρ* > 0.05; *Ρ ≤ 0.05; **Ρ ≤ 0.01).

Relative to the unsuppressed response to LPS (Fig. 4, ‘no targets’), the interaction of macrophages with apoptotic target cells elicited immunosuppression, independent of animal age (as in Fig. 1A). For example, at a T: R ratio of 6: 1 (Fig. 4B), expression of TNFα was suppressed by 68%. Macrophages in continual contact with apoptotic targets (Fig. 4, ‘not removed’) remained refractory to pro‐inflammatory stimulation, regardless of animal age. On the other hand, for macrophages from ‘young’ animals, the suppression conferred by apoptotic cells began to wane immediately after the removal of unengulfed targets and was notably abated 3 h after target removal [Again, taking macrophages at T: R = 6: 1 for discussion (Fig. 4B), immediately after target removal, the suppressive effect in ‘young’ macrophages was reduced by 16%, and it was decreased by another 34% 3 h after target removal). The refractory state was not lost completely for many further hours; macrophages did recover full responsiveness within 24 h (data not shown). The more pronounced apoptotic suppressive effects induced at higher T: R ratios also were more persistent (compare Fig. 4A–C). It is worth emphasizing that the recovery of responsiveness to TLR engagement following apoptotic cell interactions provides confirmation of the continued viability of responding cells.

In contrast to these results, the lifetime of the immunosuppressed state induced by apoptotic targets was greatly extended in macrophages from older animals. There was little immediate loss of suppression upon target removal. Using ‘old’ macrophages at T: R = 6: 1, for example, (Fig. 4B), immediately after target removal, the refractory effect was lessened by only 3%, and it was by eased only another 15% 3 h after target removal.

Thus, whereas our findings demonstrate that, in macrophages, there is no discernible aging‐associated alteration in the induction of immunosuppression by apoptotic targets, there is a considerable aging‐associated alteration in the persistence of their immunosuppressed state. Macrophages from older animals appear less able to attenuate the immunosuppression triggered by apoptotic target interactions; they become relatively locked into that immunosuppressed state. Calculating initial rates of recovery from determinations of TNFα expression at early times following apoptotic target removal (see Fig. 4; eighteen independent determinations within each age cohort, analysis by linear regression), we estimate the half‐life of the refractory state in ‘young’ mice to be approximately 1.9 h, while it is prolonged to 6.1 h in ‘old’ mice. The persistence of the response triggered by apoptotic cells suggests that macrophages from older animals have diminished flexibility in their range of responsiveness and might be less able to respond nimbly to new immune challenges.

### Impaired macrophage TLR response termination with age

We wondered whether the aging‐impaired termination of IAI responsiveness might reflect a more general aging‐associated alteration in immune response dynamics and homeostatic regulation. In this context, we chose to examine an opposing, pro‐inflammatory response.

We took advantage of the same pro‐inflammatory TLR4‐dependent LPS stimulation utilized throughout this study. Whereas it was relatively straightforward to examine the lifetime of the immunosuppressed state, it was not so simple to evaluate the response dynamics of the pro‐inflammatory state. We had assessed the persistence of IAI suppression kinetically by removing the apoptotic stimulus and measuring the appearance of [intracellular] TNFα above the suppressed, TNFα‐negative background (Fig. 4). In contrast to this sensitive ‘gain‐of‐function’ assay, a comparable evaluation of the end of the pro‐inflammatory state, involving the expeditious removal of the LPS stimulus and the quantification of the loss of TNFα protein accumulation, was not feasible. The thorough, timed removal of LPS cannot be accomplished effectively, and the fixation of permeabilized cells, necessary for reliable intracellular immunostaining, compromises protein turnover and, consequently, normal cytokine dynamics. We took an alternative approach, evaluating LPS‐dependent transcription on the population level following interruption of proximal TLR4 signaling with the small molecule inhibitor, TAK‐242 (Kawamoto *et al*., 2008).

**Figure 5:**
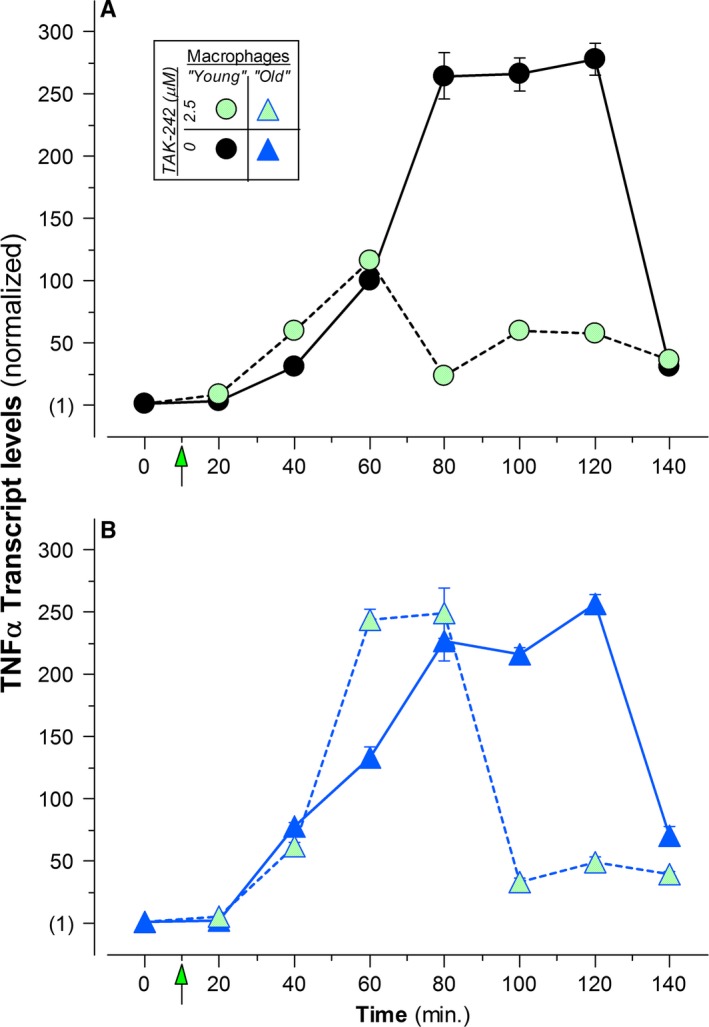
Macrophage TLR response termination is impaired with age. A representative example of the analysis of TNFα transcript levels in elicited peritoneal macrophages from individual ‘young’ (A; 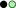) and ‘old’ (B; 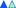) C57BL/6 mice, following incubation with LPS (5 ng mL^−1^) alone, beginning at t_0_ (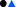), or with LPS, beginning at t_0_, and TAK‐242 (2.5 μm), beginning at *t*_10 min_ (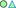, TAK‐242 addition is indicated with arrows). TNFα transcript levels were assessed by RT–qPCR analysis and were normalized for the 18S rRNA content of each sample and normalized further to TNFα mRNA levels of uninduced macrophages of the same mouse. Values for LPS response persistence (Table 1) were derived from a compilation of these analyses, as described.

The data in Fig. 5, which are taken from one representative experiment, show the rapid induction of TNFα transcripts following the addition of LPS (at *t*_0_), reaching maximum levels within about 1 h, the sustained maintenance of maximally induced levels of TNFα transcripts for almost another hour, and the subsequent diminution of TNFα transcript levels reflective of feedback regulation associated with endotoxin tolerance and the normal half‐life of TNFα transcripts (*t*_1/2_ ≅ 20 min; data not shown). This pattern is shared by macrophages from ‘young’ (Fig. 5A) and ‘old’ (Fig. 5B) animals. Consistent with our immunostaining and multiplex analyses of cytokine expression [Fig. 1, also see (Pattabiraman *et al*., 2016)], basal and maximally induced TNFα transcript levels do not vary as a function of animal age.

These data also document LPS response termination revealed upon interference specifically with TLR4 signaling by TAK‐242 (10 min after LPS addition). This treatment allows normal TLR4‐dependent TNFα induction to ensue and leads to the decline in TNFα transcript levels independent of LPS‐driven feedback regulation. For macrophages from ‘young’ mice (Fig. 5A), the decline began about 50 min after TAK‐242 addition, even before the attainment of fully induced transcript levels. Notably, for macrophages from ‘old’ animals (Fig. 5B), the decline in transcript levels began significantly later, after about 70 min and after fully induced transcript levels had been achieved. TAK‐242 treatment exerted similar effects on IL‐1β and IL‐6 transcript levels (Table 1). Combining multiple independent analyses and defining the persistence of the LPS response as the time after the addition of TAK‐242 until maximally achieved inflammatory cytokine mRNA levels begin to decline, it is clear that LPS response persistence in macrophages is prolonged considerably with aging (Table 1).

**Table 1:** Aging alters the persistence of LPS‐induced pro‐inflammatory responses in macrophages

	LPS response persistence			
	‘Young’	‘Old’	Aging‐associated prolongation (%)	*P*
	[min (mean ± SEM)]			
*TNF*α	57.3 ± 2.7	70.3 ± 2.8	22.7	*
*IL‐1*β	40.3 ± 1.8	70.3 ± 1.5	74.4	***
*IL‐6*	48.2 ± 0.2	74.4 ± 2.9	54.5	***

The persistence of the LPS response, defined here for a particular pro‐inflammatory cytokine as the time after the addition of TAK‐242, an inhibitor of proximal TLR4 signaling, until maximally achieved levels of that cytokine mRNA begin to decline, was assessed in elicited C57BL/6 macrophages isolated from mice of 2–3 months of age (‘young’) and mice of 24–25 months of age (‘old’). Macrophages isolated from individual ‘young’ (*n* = 5) and ‘old’ mice (*n* = 5) were left unstimulated, were stimulated with LPS (5 ng mL^−1^), or were stimulated with LPS and additionally treated with TAK‐242 (2.5 μm) 10 min after the addition of LPS. Cytokine transcript levels were assessed by RT–qPCR analysis. A representative example of the analysis for TNFα transcript levels in macrophages from individual ‘young’ and ‘old’ mice is presented in Fig. 5. Values for response persistence were derived by fitting the data (relative transcript levels vs. time) from individual analyses to interpolating cubic spline curves and solving for *t*_max_. The significance of differences between age groups, as calculated by Student's *t*‐test, is indicated (*Ρ ≤ 0.05; ***Ρ ≤ 0.001).

Just as in the case of responsiveness to apoptotic targets, these results suggest that aging is associated with nuanced differences in the dynamics of macrophage responsiveness to TLR signaling. It is remarkable that the aging‐associated alteration of TLR response termination, seen on the transcript level (Fig. 5), parallels the effect of aging on the persistence of IAI immunosuppression, observed on the level of protein accumulation (Fig. 4). With advancing age, it appears that the homeostatic immune regulation of macrophages, and their consequent response flexibility, is impeded generally. We do not yet know whether a discrete age exists for the onset of this phenomenon. Our preliminary analysis of LPS response termination in ‘middle‐aged’ mice suggests that enhanced persistence is evident already by 15 months of age (data not shown).

## Discussion

Our exploration of macrophage responsiveness to apoptotic cells as a function of animal age has led to striking results. The varied and extensive immune dysregulation associated with aging has prompted interest in the identification of causal defects on the cellular level. We had shown previously that the magnitude of TLR‐dependent responsiveness in murine macrophages is not altered generally with age (Pattabiraman *et al*., 2016). Here, we demonstrate that the magnitude of IAI responsiveness in murine macrophages also does not change as a function of animal age. In contrast, we found an aging‐associated alteration in the termination of IAI. More generally, we have identified a cell‐intrinsic alteration in the homeostatic resolution of macrophage immune responsiveness that arises with age. This alteration may be an early, causal event in the imbalanced pathology of immunosenescence.

As apoptotic cells potently modulate immune responsiveness, we had hypothesized that the ability of apoptotic cells to elicit innate apoptotic immune responses or the magnitude of IAI responsiveness itself might be altered in an aging‐associated manner. In fact, our data demonstrate that aging does not affect the immunoregulatory potency of apoptotic cells or the magnitude of apoptotic responsiveness. Rather, the immunosuppressed period triggered by apoptotic targets, which is relatively brief in macrophages from ‘young’ mice, is prolonged over threefold in macrophages from older animals. The vigorous and relatively short‐lived anti‐inflammatory state of peritoneal macrophages from ‘young’ mice underscores the homeostatic context in which robust responses are coupled to prompt restoration of the resting state. Aging‐associated response persistence reflects a cell‐intrinsic alteration of responding macrophages. Just as IAI serves an important role in dampening pro‐inflammatory immune responses, we envision that the transience of its dampening effect is significant, facilitating the dynamic flexibility of cellular responsiveness.

While our hypothesis that IAI responsiveness is altered with animal age was satisfied by these observations, we found that the aging‐associated alteration in response termination is not limited to IAI. In contrast to the absence of an aging‐associated alteration in the initiation or magnitude of pro‐inflammatory responses triggered by LPS, or other TLR agonists (Pattabiraman *et al*., 2016), the lifetime of those responses, too, is prolonged with age. Whereas the flexible responsiveness of macrophages facilitates their sensitive role as environmental sentinels, aging‐impeded response termination represents a reduction of homeostatic regulation and likely impacts the nimbleness of their responsiveness. This aging‐associated alteration in the nimbleness with which macrophages are able to alternate between opposing responses may be of particular significance to hyperinflammatory conditions associated with immunosenescence. Among the most characteristic attributes of immunosenescence is the elevated inflammatory status of many individuals in the absence of overt infection. We speculate that increased inflammation without obvious infection may represent a record of past immunological challenge. It is interesting that the aging‐associated decline in immune response termination appears in middle age, before pathological manifestations of immunosenescence are evident. We speculate that aging‐associated alterations in macrophage homeostatic control may affect immune responses generally and are causally involved in immune decline and the pathology of immunosenescence.

The molecular mechanism of the aging‐associated alteration in response dynamics remains to be determined and is a critical topic of future work. Our observations point strongly toward a common aging‐associated effect exerted on or upstream of the level of transcription. Indeed, although we have employed various measures in the experiments reported here, both IAI and TLR‐dependent responses are manifest primarily on the level of transcription. Changes in cytokine transcript stability are unlikely to be responsible, as we have observed this effect regardless of the particular cytokine assessed (Table 1) and the stability of cytokine transcripts is not altered generally with age (Frasca *et al*., 2012). Similarly, because signal transduction involved in IAI is distinct and independent of TLR‐dependent (including endotoxin tolerance‐related) signaling (Cvetanovic & Ucker, 2004; Cvetanovic *et al*., 2006; Ucker, 2009), it is unlikely that any one signaling component is affected. It may be that common effectors of signaling molecule regulation (e.g., protein phosphatases) are at play. Alternatively, we speculate that the process of chromatin remodeling impacting transcriptional accessibility may be involved. The role of chromatin‐modifying transcriptional coregulators has been implicated previously in IAI responsiveness (Cvetanovic & Ucker, 2004). A central role for chromatin structure – and chromatin remodeling enzymes – in aging has been proposed (Pegoraro & Misteli, 2009; Das & Tyler, 2012). Certainly, with advancing age, the possibility of diminished chromatin restoration, such as effected by energy‐dependent, sirtuin‐like histone deacetylases, provides a compelling mechanistic paradigm for altered response dynamics.

In this study, we focused attention on primary macrophages, as critical effectors of innate immunity. The work presented describes studies with elicited peritoneal macrophages (for which recoverable numbers of responsive cells made extensive surveys feasible); completely comparable results were obtained with resident peritoneal and spleen macrophages. It remains to be tested whether the aging‐associated alteration in homeostatic regulation that we observed is evident in myeloid and other cell types distinct from macrophages (especially dendritic cells), as well as in comparable human cells. It also will be fascinating to extend these analyses to adaptive immune and other, nonimmune responses.

The novel and intriguing findings presented here, highlighting nuanced aging‐associated dynamic alterations evident at the cellular level, prompt a reconsideration of expectations for changes that may underlie immunosenescence. No clear mechanistic defects have been identified to date that are obviously responsible for the variety of alterations associated with immunosenescence. We propose that aging‐ and immunosenescence‐associated alterations in immune function observed organismally may reflect an underlying, cell‐intrinsic aging‐associated impairment in homeostatic recovery following immune stimulation, and a consequent diminution in the cellular nimbleness of immune responsiveness.

## Experimental procedures

Methodological information is included in Data S1 (Supporting information).

## Funding

This work was supported in part by NIH grant AG029633 to DSU.

## Conflict of interest

The authors declare that they have no conflicts or competing commercial interests in relation to this work.

## Author contributions

GP planned, undertook, and analyzed all of the experiments; KP performed some of the cell culture and RT–qPCR analyses; JPG developed the cytofluorimetric methods employed; DSU conceived the project, and oversaw and analyzed the studies; GP and DSU wrote the manuscript.

## Supporting information


**Fig. S1** Aging does not alter the magnitude of IAI responsiveness of Balb/cBy macrophages.

**Fig. S2** Serum cytokine concentrations reveal aging‐associated imbalances that typify immunosenescence.

**Fig. S3** Macrophage IAI responses are not dependent upon apoptotic cell engulfment.

**Fig. S4** Macrophage IAI responsiveness with thymocyte targets.

**Fig. S5** Aging does not alter the target cell phagocytic activity of macrophages.

**Data S1** Experimental procedures.
