# Supplementary figures and images for: Aging‐associated dysregulation of homeostatic immune response termination (and not initiation)
Source: Aging Cell. 2017 Mar 30;16(3):585–93. doi: 10.1111/acel.12589 (PMC5418197; doi:10.1111/acel.12589)

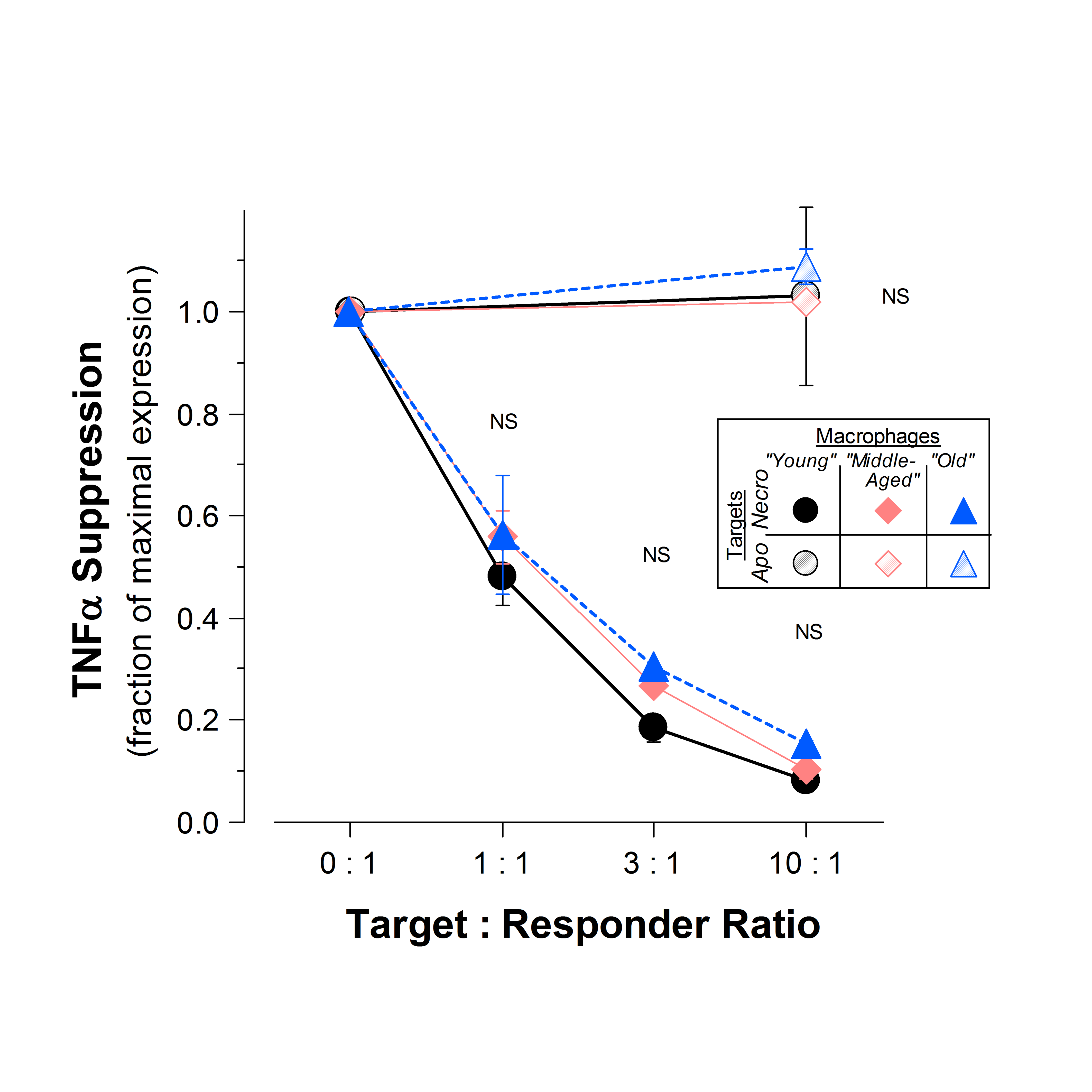

Supplement: Supplementary file 1 — Fig. S1 Aging does not alter the magnitude of IAI responsiveness of Balb/cBy macrophages. [file ACEL-16-585-s001.tif]

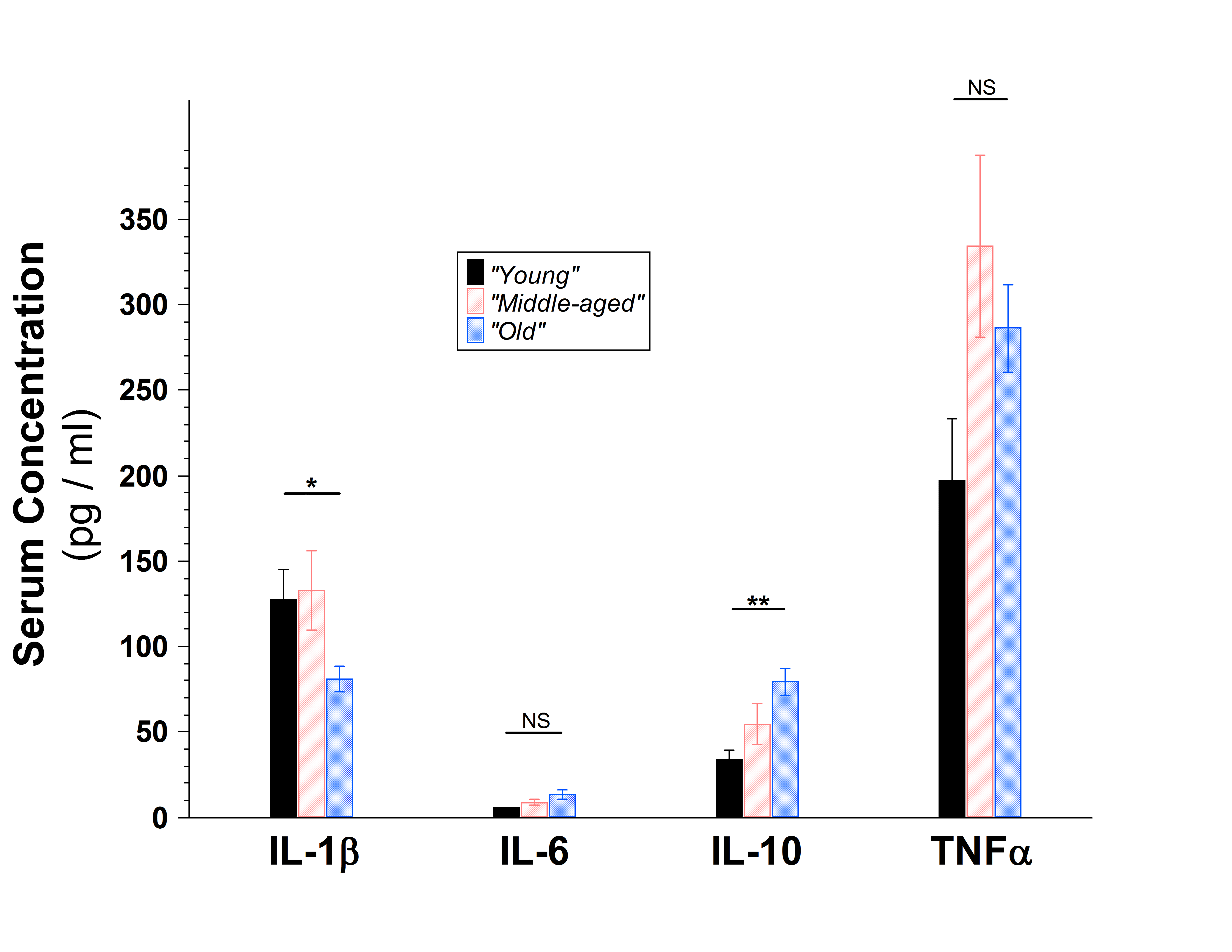

Supplement: Supplementary file 2 — Fig. S2 Serum cytokine concentrations reveal aging‐associated imbalances that typify immunosenescence. [file ACEL-16-585-s002.tif]

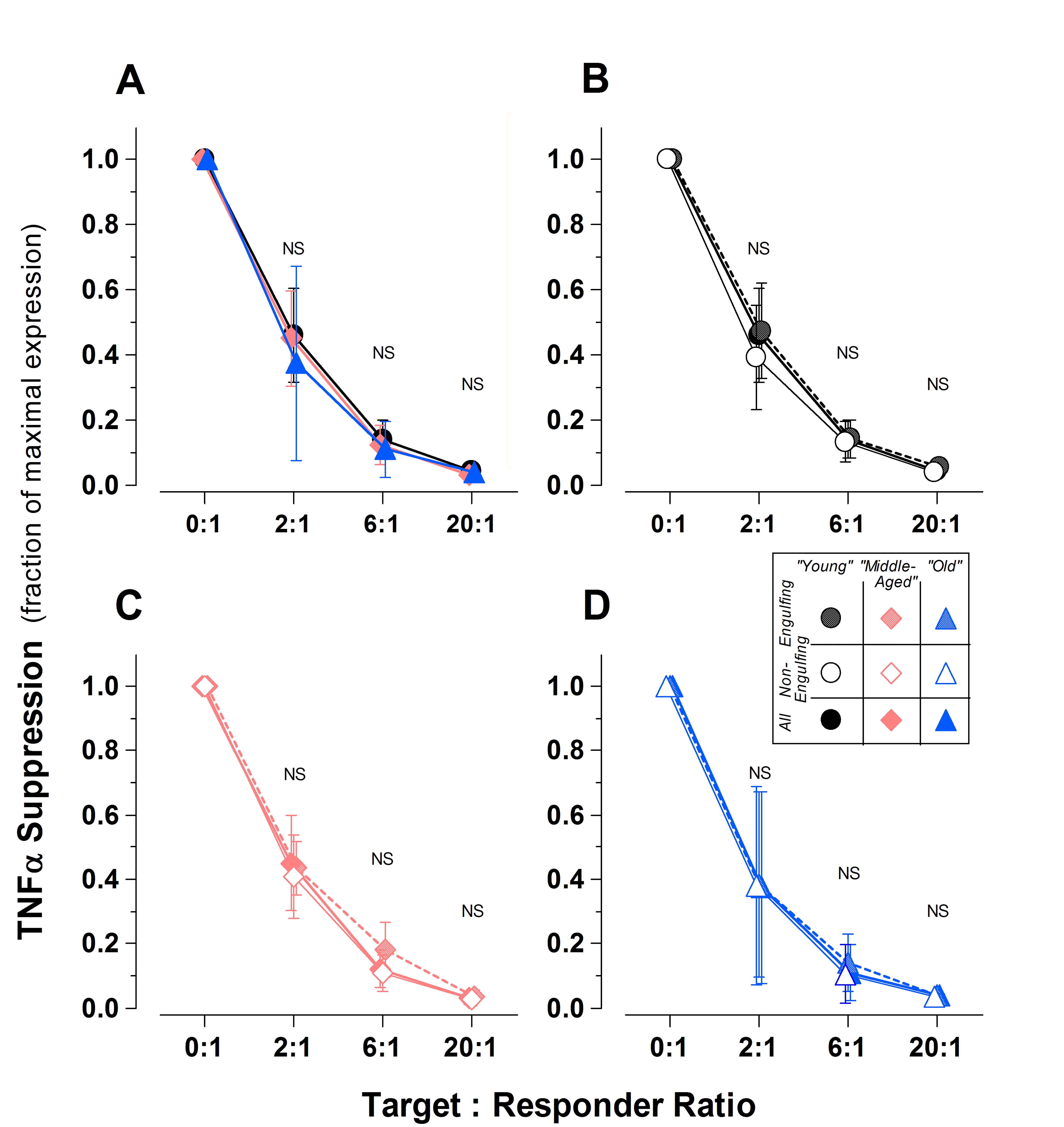

Supplement: Supplementary file 3 — Fig. S3 Macrophage IAI responses are not dependent upon apoptotic cell engulfment. [file ACEL-16-585-s003.tif]

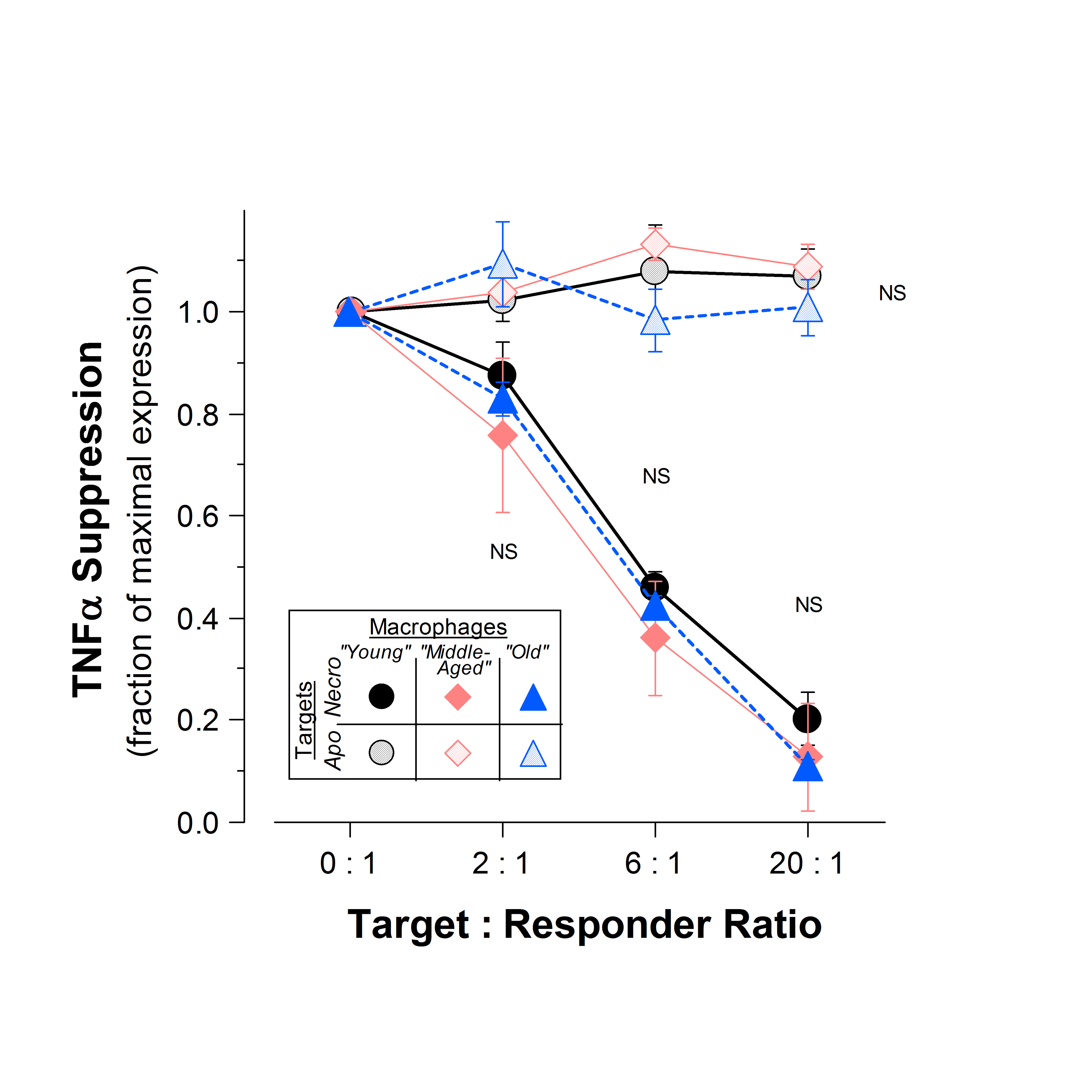

Supplement: Supplementary file 4 — Fig. S4 Macrophage IAI responsiveness with thymocyte targets. [file ACEL-16-585-s004.tif]

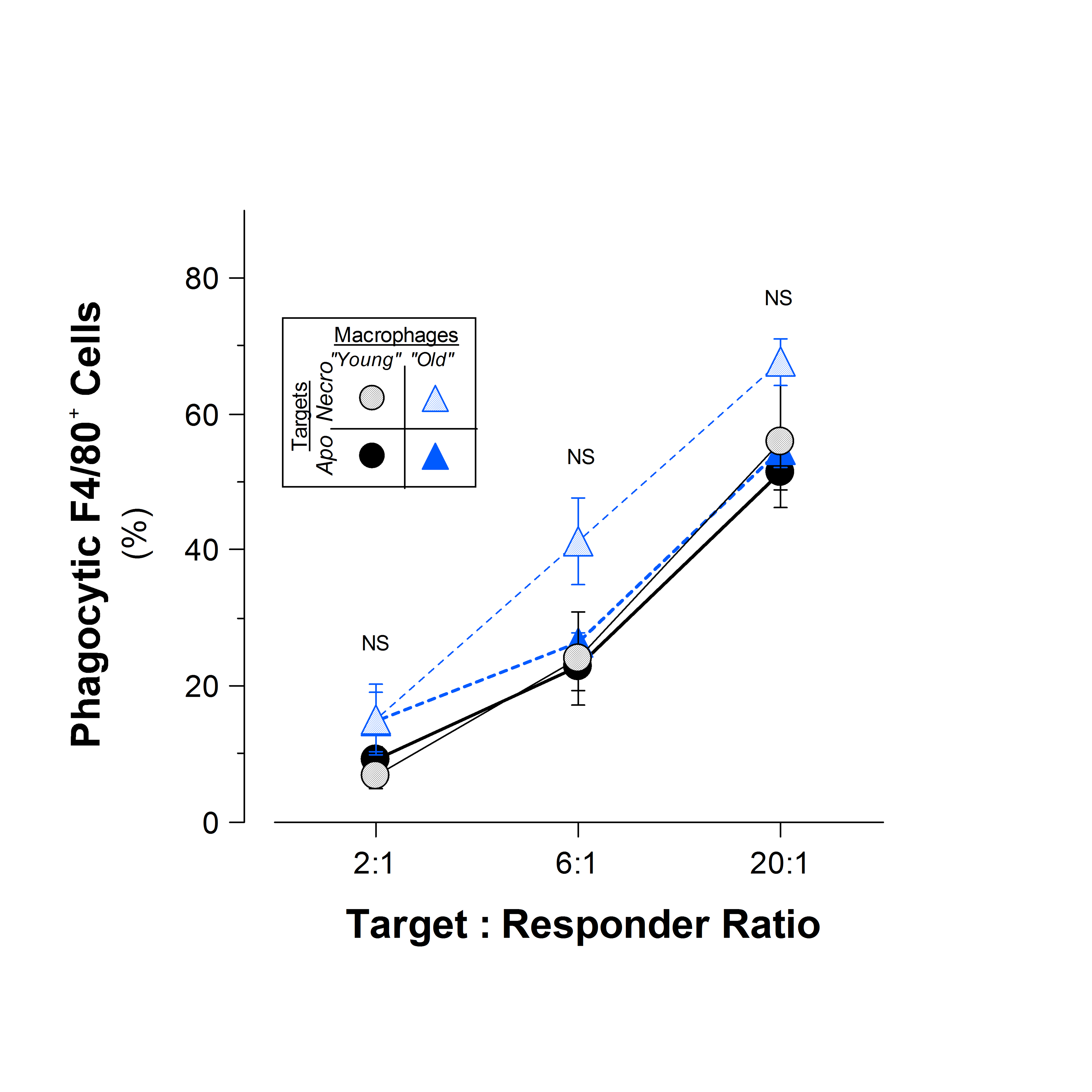

Supplement: Supplementary file 5 — Fig. S5 Aging does not alter the target cell phagocytic activity of macrophages. [file ACEL-16-585-s005.tif]
