# Supplementary material for: Aging‐associated dysregulation of homeostatic immune response termination (and not initiation)
Source: Aging Cell. 2017 Mar 30;16(3):585–93. doi: 10.1111/acel.12589 (PMC5418197; doi:10.1111/acel.12589)
Supplement: Supplementary file 6 — Data S1 Experimental procedures. [file ACEL-16-585-s006.doc]

**Supporting Information**

**Experimental Procedures**

**Analytical approach**

In order to evaluate intrinsic macrophage function, we used sensitive and specific multiparameter cytofluorimetric analyses. We evaluated quantitatively, on the level of the single cell, macrophage responsiveness to pro-inflammatory stimulation (via TLR 4 with *E. coli* lipopolysaccharide [LPS]) and to anti-inflammatory modulation by exogenous syngeneic apoptotic cells (especially spleen cells induced to die by treatment with staurosporine). Macrophages were identified with an antibody specific for F4/80, a definitive macrophage marker, to gate on F4/80^+^ cells. (Macrophages also could be discriminated by scatter properties; data not shown.) We analyzed the expression and intracellular presence in macrophages of the pro-inflammatory cytokines TNFα. Complementarily, we analyzed the accumulation of the anti-inflammatory cytokine IL-10. Cytokine secretion was blocked with Brefeldin A, an inhibitor of *trans*-Golgi transport, in order to visualize cytokine expression in cells individually; cells were immunostained intracellularly following permeabilization with saponin and fixation. Importantly, the Brefeldin A treatment used (3 hr., 5 μg/ml) is not lethal for these cells (data not shown), and traps cytokines intracellularly, generating clearly distinct subpopulations of cells that do or do not express the relevant cytokine, and that can be quantified readily (see **Fig. 1B-D**). (We find that IL-10 accumulates intracellularly even without the blockade of secretion, although Brefeldin A does not interfere with the analysis of its expression; data not shown.) Apoptotic cells were labeled differentially, enabling us to assess their phagocytosis by macrophages. For brevity, we refer to apoptotic cells as “targets” and to macrophages as “responders” throughout this manuscript.

**Mice**

C57BL/6 and similarly long-lived Balb/cBy mice (of both genders) of discreet ages spanning their normal adult lifespan (young adults of 2 - 3 months of age [referred to as “young”], middle-aged adults of 15 months of age [“middle-aged”], and older adults nearing the end of mean lifespan [24 - 25 months of age; termed “old”]) were obtained from the National Institute of Aging (Bethesda, MD). The mean lifespan of these mice is about 26 months (see http://research.jax.org/faculty/harrison/ger1vi_LifeStudy1.html). All mice were housed in a single environmentally-controlled room within the UIC animal facility. It is important to note that our studies focused on animals of a single colony (and housed together), with age as the single variable. (Observations reported here have been confirmed with several cohorts of aged mice over the course of several years.) All animal experiments and procedures were approved by the UIC Animal Care and Use Committee.

**Reagents**

*Antibodies:* Pacific Blue (PB) - conjugated F4/80-specific rat monoclonal antibody (mAb) was obtained from Invitrogen Corporation (Frederick, MD). Phycoerythrin (PE) - conjugated TNFα-specific rat mAb and IL-10-specific rat mAb conjugated with phycoerythrin/cyanine (PE/Cy7) tandem dye were obtained from BioLegend Inc. (San Diego, CA).

*Other reagents, inhibitors, and drugs:* All primers were purchased from Integrated DNA Technologies, Inc. (Coralville, IA). Staurosporine (from *Streptomyces* sp.), Lipopolysaccharide (LPS; from *E. coli* 011:B4), 5(6)-carboxyfluorescein diacetate N-succinimidyl ester (CFDA), and Brefeldin A (from *Penicillium brefeldianum*) were purchased from Sigma-Aldrich. TAK-242 (CLI-095), a cell permeable TLR4 signaling inhibitor (also known as CLI-095) was purchased from InvivoGen (San Diego, CA).

**Cell Culture and Death Induction**

Primary murine splenocytes, thymocytes, and macrophages (from the C57BL/6 mice described above, unless otherwise noted), and DO11.10 murine T hybridoma cells (Cvetanovic & Ucker 2004) were cultured at 37°C in a humidified 5% (v/v) CO_2_ atmosphere in RPMI 1640 medium (Mediatech, Herndon, VA) supplemented with heat-inactivated FBS (10% v/v; HyClone Laboratories, Logan, UT), 2 mM L-glutamine, and 50 µM 2-mercaptoethanol. (This supplemented medium is referred to below as “complete RPMI medium”).

Physiological cell death (apoptosis) was induced by treatment of cells with actinomycin D (200 ng/ml; 12 hr.) or by treatment with staurosporine (1 µM in serum-free medium, 3 hr.), and was verified cytofluorimetrically (Cocco & Ucker 2001). Pathological cell death (necrosis) was triggered by incubation of cells at 56°C for 20 min. (until trypan blue uptake indicated compromise of membrane integrity). In all cases, target cells (viable, apoptotic, and necrotic cells) were washed four times in complete RPMI medium before experiments. Targets were prepared from splenocytes or thymocytes taken from “young” mice, unless otherwise noted.

**Isolation of Macrophages**

Macrophages from individual mice were analyzed separately. Where indicated, results are derived from the compilation of multiple analyses of macrophages from individual mice.

*Thioglycollate-Elicited Peritoneal Macrophages:* Thioglycollate (2 ml of 4% thioglycollate broth; Difco; Irvine, CA) was injected intraperitoneally. Three days later, elicited cells were collected by flushing the peritoneal cavity with 5 ml complete RPMI medium. Recovered cells were washed twice in complete RPMI medium, plated in 100 mm dia. dishes, and allowed to adhere for 3 - 4 hours. Adherent cells were washed twice more in complete RPMI medium, collected, and re-plated (0.5 × 10^6^ cells/ml/well) in wells of 24-well plates. (For cytokine secretion studies, macrophages were plated at 0.2 × 10^6^ cells/ml/well.) With this procedure, we were able to recover as many as 1 × 10^7^ macrophages per mouse.

**Assessment of Phagocytosis**

Phagocytosis was assessed as previously described (Cocco & Ucker 2001; Cvetanovic & Ucker 2004). Target cells were labeled green with 5(6)-carboxyfluorescein diacetate (CFDA) *N*-succinimidyl ester (0.2 μM), and then were induced to undergo apoptotic cell death, killed pathologically, or left untreated. Macrophages were co-cultured with apoptotic, necrotic, or viable target cells for 2 hr. at 37°C. Macrophages then were washed with PBS, followed by a wash with PBS supplemented with 0.4 mM Na_2_EDTA and a wash with 0.05% trypsin-EDTA to remove any bound, unengulfed targets. Cells were gently lifted by scarping, stained with PB - conjugated F4/80 antibody (to identify macrophages), and analyzed cytofluorimetrically. Cells with macrophage-like scatter properties that stained positively for F4/80 and also were CFDA-positive represented macrophages that had engulfed targets. Phagocytosis is represented as the fraction of F4/80^+^ macrophages that are CFDA^+^. Most targets that are bound but not engulfed are disrupted and do not remain adherent during the analysis.

**Cytofluorimetric Analyses**

For extra-cellular staining with primary antibodies conjugated to PB, PE, or PE-Cy7, cells were washed twice with cold PBS containing FBS (1%) before resuspension and staining in this same buffer for 25 min. at 4°C in the dark prior to washing and cytofluorimetric analysis. For intracellular staining, cells were washed twice with cold PBS and fixed and permeabilized in a solution of 4% formaldehyde and 0.1% saponin in PBS for 20 min. at 4°C in the dark. After fixation, cells were washed twice with PBS buffer containing 0.1% saponin and 1% FBS and stained in this same buffer. In the case of intracellular cytokine detection, the normal process of secretion was blocked by treatment of cells with Brefeldin A (5 µg/ml), for 3 hr. prior to fixation and permeabilization of cells. Cells were analyzed cytofluorimetrically on FACSCalibur or the BD LSRFortessa instruments (BD Biosciences, San Jose CA). Cytofluorimetric data were processed with Summit version 4.3 software (Dako, Carpentaria, CA).

**Quantification of Cytokine Release**

Quantification of serum cytokine levels and secreted cytokines levels were measured using Bio-Plex Multiplex System (Bio-Rad Life Sciences, Hercules, CA) as per manufacturers’ protocol. Briefly, samples were diluted as necessary (serum samples in the provided standard diluent, and the cell culture supernatants in culture media). 50µl of samples were loaded in each well containing anti-cytokine specific beads for the multiplex assay. Following incubation and wash steps, the cytokine levels were determined by probing with Bio-Plex Detection Antibody, and followed by Streptavidin-PE. After washing wells, the plate was read using the Bio-Plex 200 System (Bio-Rad) set to read 100 beads/region and cytokine levels were analyzed and quantified using Bio-Plex Manager software (Bio-Rad).

**RNA Extraction, Reverse Transcription - Quantitative Polymerase Chain Reaction (RT-qPCR) Analysis**

Total RNA was isolated using TRIzol reagent (Invitrogen, Carlsbad, CA) and treated with DNase to remove contaminating DNA, using TURBO DNA-free (Ambion, Life Technologies Corporation, Grand Island, NY). (All procedures involving commercial products followed the manufacturers’ protocols.) cDNA synthesis (starting with 2 µg of total RNA) was performed with random hexamers using High Capacity cDNA Reverse Transcription Kit (Applied Biosystems, Life Technologies Corporation; Grand Island, NY). Reverse Transcription – Quantitative Polymerase Chain Reaction (RT-qPCR) reactions were performed using Fast SYBR green (Applied Biosystems) and run on the ViiA™ 7 RealTime PCR System (Invitrogen). Primer pairs (listed below) were designed to hybridize with murine-specific sequences exclusively, and to prime cDNA synthesis spanning exons, thereby minimizing the background of cDNA synthesis from contaminating genomic DNA. Standard curves for 18S rRNA, TNFα, IL-6, and IL1β were generated using cDNA templates prepared from cells stimulated with LPS alone. Amplifications for each RT-qPCR primer/probe set were calibrated by linear regression according to the formula: CN = 10^-[(TC -^ *^b^*)/*^m^*], where CN is the relative template copy number, TC is the observed threshold crossing point, and *b* and *m* are intercept and slope constants, respectively. Experimental cDNA samples were diluted as necessary to yield RT-qPCR results within the linear range of the relevant standard curve. Samples were normalized relative to their 18S rRNA content, determined in parallel RT-qPCR reactions; relative transcript concentrations were calculated assuming a constant efficiency of cDNA synthesis. For the comparison of two samples (treated [*t*] and untreated [*u*], with the untreated sample serving as a further normalization control), CN*_t_*/CN*_u_* = {10^[-(TC^*^t^* - *^b^*)/*^m^*]}/{10^[-(TC^*^u^* - *^b^*)/*^m^*]}, and log (CN*_t_* - CN*_u_*) = (TC*_u_* - TC*_t_*)/*m*.

Primers (sequences are 5’⇨3’):

18S rRNA

*Forward:* CCGCAGCTAGGAATAATGGA

*Reverse:* CCCTCTTAATCATGGCCTCA

TNFα

*Forward:* CAAATGGCCTCCCTCTCAT

*Reverse:* CTCCTCCACTTGGTGGTTTG

IL-1β

*Forward:* TGAAGTTGACGGACCCCAAA

*Reverse:* GCTCTTGTTGATGTGCTGCT

IL-6

*Forward:* TACCACTCCCAACAGACCTG

*Reverse* CAAGTGCATCATCGTTGTTCA

**Statistical Analyses**

The data values presented are the means of replicate determinations, and error bars represent the Standard Error of the Mean [SEM]. Statistical analysis comparing the values of two experimental sample sets involved 2-tailed Student’s t-test. Statistical analysis of multiple data sets, involving a single variable (e.g. age), utilized 1-way Analysis of Variance (ANOVA); where two different variables (e.g. age and dose response) are involved, 2-way ANOVA was employed. The significance of differences was corrected for multiple comparisons by the Bonferroni method (with a significance level [α] of 0.05). Where indicated (*: ρ ≤ 0.05; **: ρ ≤ 0.01; ***: ρ ≤0.001), results allowed the rejection of a null hypotheses [that results from different age cohorts were not different]; “NS” denotes cases in which differences between sample sets were not statistically significant (ρ > 0.05).

**Supplemental Figures**

**Supplemental Figure 1. Aging does not alter the magnitude of IAI responsiveness of Balb/cBy macrophages.**

The magnitude of apoptotic suppression of TNFα expression in macrophages isolated from Balb/cBy mice of 2 - 3 months of age (“young”,), mice of 15 months of age (“middle-aged”; ) and mice of 24 - 25 months of age (“old”; ) was determined, as in **Figure 1A**. Elicited peritoneal macrophages from individual mice within each age cohort were cultured with apoptotic (“apo”; actinomycin D-treated; ) or necrotic (“necro”; heat-killed;) DO11.10 murine T hybridoma cell targets at the indicated target : responder (T : R) ratio and / or LPS (5 ng / ml) for 5 hr. Brefeldin A was included during the last 3 hr. and staining and MFI-based result calculations were performed as in **Figure 1A**. The data presented are compiled from the results of the analysis of macrophages from 3 individual mice within each age cohort. While the suppression of TNFα expression was elicited specifically by apoptotic (and not necrotic) targets, there were no statistically significant differences among age groups with respect to responsiveness, as calculated by 1-way ANOVA. There also were no statistically significant differences among age groups treated or untreated with necrotic targets, as calculated by 2-way ANOVA.

**Supplemental Figure 2. Serum cytokine concentrations reveal aging-associated imbalances that typify immunosenescence.**

The serum concentrations of the indicated cytokines in individual “young” (), “middle-aged” (), and “old” () C57BL/6 mice were determined by multiplex immunoassays. The data presented are compiled from the results of the analysis of 18 individual mice within each age cohort. The significance of differences among age groups, with respect to the particular cytokine assessed, as calculated by 1-way ANOVA, are indicated (NS: ρ > 0.05; *: ρ ≤ 0.05; **: ρ ≤ 0.01).

**Supplemental Figure 3. Macrophage IAI responses are not dependent upon apoptotic cell engulfment.**

The magnitude of apoptotic suppression of TNFα expression was determined in macrophages that had and had not engulfed apoptotic targets. Macrophages were isolated from C57BL/6 mice of 2 - 3 months of age (“young”;; 4 mice of this age cohort were analyzed individually), mice of 15 months of age (“middle-aged”;; 3 mice of this age cohort were analyzed individually) and mice of 24 - 25 months of age (“old”; ; 3 mice of this age cohort were analyzed individually). Elicited peritoneal macrophages from individual mice within each age cohort were cultured with apoptotic targets (CFSE-labeled splenocytes prepared from “young” syngeneic mice, treated with staurosporine) at the indicated target : responder (T : R) ratio and / or LPS (5 ng / ml) for 5 hr. Brefeldin A was included during the last 3 hr. Staining and result calculations were performed as in **Figure 1A**. Macrophages that had engulfed targets were identified as CFSE^+^ F4/80^+^ cells; CFSE^-^ F4/80^+^ cells were considered to be non-engulfing macrophages. Filled symbols () denote TNFα expression in the whole population of macrophages (**Panel A**). In **Panels B** (“young”), **C** (“middle”), and **D** (“old”), cross-hatched symbols (, with dashed lines) indicate TNFα expression in engulfing macrophages, and open symbols (, with thin solid lines) denote TNFα expression in non-engulfing macrophages. Filled symbols (, with thick solid lines, as in **Panel A**) again indicate TNFα expression in the whole population of macrophages of the respective age cohort. There were no statistically significant differences (NS: ρ > 0.05) among age groups with respect to responsiveness, as calculated by 1-way ANOVA, or between engulfing and non-engulfing macrophages within each age cohort, as calculated by Student’s t-test.

**Supplemental Figure 4. Macrophage IAI responsiveness with thymocyte targets.**

The magnitude of apoptotic suppression of TNFα expression in macrophages isolated from C57BL/6 mice of 2 - 3 months of age (“young”,), mice of 15 months of age (“middle-aged”;) and mice of 24 - 25 months of age (“old”;) was determined with syngeneic thymocyte targets. Elicited peritoneal macrophages from individual mice within each age cohort were cultured with apoptotic (“apo”; staurosporine-treated;) or necrotic (“necro”; heat-killed;) thymocyte targets (prepared from “young” syngeneic mice) at the indicated target : responder (T : R) ratio and / or LPS (5 ng / ml) for 5 hr. Brefeldin A was included during the last 3 hr. and staining and MFI-based result calculations were performed as in **Figure 1A**. The data presented are compiled from the results of the analysis of macrophages from 4 individual mice within each age cohort. While the suppression of TNFα expression was elicited specifically by apoptotic (and not necrotic) thymocyte targets, there were no statistically significant differences among age groups with respect to responsiveness, as calculated by 1-way ANOVA. For necrotic targets, there were no statistically significant differences among age groups or as a function of dose, as calculated by 2-way ANOVA. Note that, while half-maximal suppression occurs at a ratio of 1.6 (± 0.12) apoptotic splenocyte targets per macrophage (**Figure 1A**), a higher dose of much smaller apoptotic thymocyte targets (5.2 ± 0.30) is needed to achieve half-maximal suppression.

**Supplemental Figure 5. Aging does not alter the target cell phagocytic activity of macrophages.**

The magnitude of phagocytosis of CFDA-labeled syngeneic apoptotic (“apo”; staurosporine-treated;) or necrotic (“necro”; heat-killed;) splenocyte targets was assessed cytofluorimetrically in elicited peritoneal macrophages isolated from “young” () and “old”
() C57BL/6 mice, as described in Experimental Procedures. The data presented are compiled from the results of the analysis of macrophages from 4 individual mice within each age cohort. There were no statistically significant differences (NS: ρ > 0.05) between age groups, as calculated by 2-way ANOVA.
